# Novel strategies for the treatment of myelofibrosis driven by recent advances in understanding the role of the microenvironment in its etiology

**DOI:** 10.12688/f1000research.18581.1

**Published:** 2019-09-19

**Authors:** Zimran Eran, Maria Zingariello, Maria Teresa Bochicchio, Claudio Bardelli, Anna Rita Migliaccio

**Affiliations:** 1 Department of Hematology, Hadassah University Center, Jerusalem, Israel; 2 Unit of Microscopic and Ultrastructural Anatomy, Department of Medicine, University Campus Bio-Medico, Rome, Italy; 3 Istituto Scientifico Romagnolo per lo Studio e la Cura dei Tumori (I.R.S.T.), IRCCS, Meldola (FC), Italy; 4 Dipartimento di Scienze Biomediche e NeuroMotorie, Alma Mater Studiorum - Università di Bologna, Bologna, Italy

**Keywords:** Myelofibrosis, p53, transforming growth factor beta, combination therapy, MDM2 inhibitors, transforming growth factor beta inhibitors, animal models, pre-clinical studies

## Abstract

Myelofibrosis is the advanced stage of the Philadelphia chromosome-negative myeloproliferative neoplasms (MPNs), characterized by systemic inflammation, hematopoietic failure in the bone marrow, and development of extramedullary hematopoiesis, mainly in the spleen. The only potentially curative therapy for this disease is hematopoietic stem cell transplantation, an option that may be offered only to those patients with a compatible donor and with an age and functional status that may face its toxicity. By contrast, with the Philadelphia-positive MPNs that can be dramatically modified by inhibitors of the novel BCR-ABL fusion-protein generated by its genetic lesion, the identification of the molecular lesions that lead to the development of myelofibrosis has not yet translated into a treatment that can modify the natural history of the disease. Therefore, the cure of myelofibrosis remains an unmet clinical need. However, the excitement raised by the discovery of the genetic lesions has inspired additional studies aimed at elucidating the mechanisms driving these neoplasms towards their final stage. These studies have generated the feeling that the cure of myelofibrosis will require targeting both the malignant stem cell clone and its supportive microenvironment. We will summarize here some of the biochemical alterations recently identified in MPNs and the novel therapeutic approaches currently under investigation inspired by these discoveries.

## Introduction

Overt myelofibrosis (MF) is the final stage of several disease entities collectively referred to as the Philadelphia chromosome-negative myeloproliferative neoplasms (MPNs) that include polycythemia vera (PV), essential thrombocythemia (ET), and prefibrotic or early stage primary myelofibrosis (pre-MF) and can also arise *de novo* as overt fibrotic-stage primary myelofibrosis (PMF) ^1, 2^ These diseases share common clinical features including constitutional and microvascular symptoms, splenomegaly, a high risk of thromboembolic and hemorrhagic complications, and a propensity to progress to a form of acute myeloid leukemia (AML) termed MPN-blast phase (MPN-BP). Early studies have identified that MPNs arise within the hematopoietic stem/progenitor cell (HSPC) compartment, and recent advances have largely elucidated its molecular pathophysiology ^3– 5^. Constitutive activation of the JAK–STAT signaling pathway driven by one of several canonical somatic mutations results in myeloproliferation and contributes to genomic instability. Acquisition of additional genetic aberrations eventually leads to disease progression ^5^. While PV, ET, and pre-MF are usually indolent hematological malignancies with a median survival spanning decades or several years, overt MF, which include PMF, carries worse prognosis and severely affects the patient’s quality of life. Usually, MPN-BP has a prognosis of only several months ^6^. The disease progression of MF exhibits a great range of patient-to-patient variability. The detailed genetic information currently available on large numbers of patients is providing evidence-based criteria for their risk stratification, which, in the future, may provide the basis for personalized therapy.

In contrast to the significant progress made in understanding the disease’s pathogenesis, treatment for MF remains largely palliative. Although we can effectively reduce symptoms and prevent thromboembolic complications, a treatment that can modify the course of the disease and prevent progression to MPN-BP is lacking. The only therapeutic option that offers potential cure is allogeneic hematopoietic stem cell (HSC) transplantation (HSCT), but this approach is limited by the lack of donors to all patients and by associated morbidity and mortality. Improving the survival of patients with MF is a major unmet need in malignant hematology. Better understanding of the pathological pathways involved in MF disease progression has ushered the development of novel treatment strategies aimed at slowing or even reversing disease progression and prolonging patient survival. An excellent review on the genetic basis of MPNs has been recently published by Vainchenker *et al*. in *F1000Research* ^5^. Here, we will summarize scientific information that is driving the search for a cure in MPNs, focusing our discussion on the most recent strategies targeting the microenvironment that are currently under investigation.

## Mutational landscape in MPNs

In 2005, four groups reported the identification of a point mutation in exon 14 of the *JAK2* gene, *JAK2*V617F, in over 95% of patients with PV and 50–60% of patients with ET and PMF ^7– 10^. JAK2 is the tyrosine kinase that represents the first signal transduction element of the receptors for erythropoietin (EPO), thrombopoietin (TPO), and granulocyte-colony stimulating factor (GSCF) ^5^. As such, JAK2 is necessary for normal hematopoietic cell growth and differentiation. *JAK2*V617 affects the inhibitory domain of the protein, rendering it constitutively active, independent of extracellular activation by the physiologic ligands. *JAK2* exon 12 mutations have later been found to drive most cases of *JAK2*V617-negative PV ^11^. Inactivating mutations in the gene encoding the TPO receptor ( *MPL*) have been identified in 3–5% of ET and PMF cases ^12^. In 2013, frameshift mutations in exon 9 of the calreticulin ( *CALR*) gene, encoding for an endoplasmic reticulum chaperone protein that interacts with MPL, were found in the majority of *JAK2*V617F and *MPL* mutation-negative ET and PMF patients, thereby completing the “missing piece in the puzzle” of MPN driver mutations ^13, 14^. In up to 10% of patients with ET and 15% of patients with PMF, a driver mutation cannot be identified. These “triple-negative” MPNs may be driven by non-canonical mutations in *JAK2* or *MPL* or by genetic lesions in other mediators of the JAK–STAT pathway such as *LNK* or *PPM1D* ^5, 15^.

Advances in genotyping, such as the application of next-generation sequencing and high-resolution chromosomal microarrays, have led to the discovery of additional somatic mutations that usually arise following acquisition of the driver mutations but can also precede them and that can contribute to disease progression and transformation to MPN-BP. These mutations affect genes involved in epigenetic regulation and splicing, such as *ASXL1*, *DNMT3A*, *TET2*, *SRSF2, U2AF1*, and *SF3B1*, as well as signaling and apoptosis, and were found across all myeloid malignancies ^5^. Mutations in *ASXL1*, *SRSF2*, *EZH2*, and *IDH1/2* have been associated with shortened survival and higher risk of progression to MPN-BP ^16^. Mutations in *U2AF1* have been associated with anemia and additional poor prognostic features ^17^. Mutations or other genetic lesions affecting the tumor suppressor p53 have been shown to play a central role in progression to MPN-BP and are highly predictive of leukemic transformation and poor outcomes ^18, 19^. The growing importance of genomic analysis in MPN patient assessment is reflected by the advent of updated risk stratification models integrating molecular and cytogenetic profiles with the more traditional clinical and morphological parameters to guide management decisions such as referral to HSCT ^20– 22^. For example, a Genetics-based International Prognostic Scoring System (GIPSS) has been proposed that is based exclusively on mutational and cytogenetic markers ^20^. Recently, comprehensive genomic characterization of 2,035 MPN patients identified distinct genetic subgroups that correlate well with clinical course and prognosis and may arguably provide more accurate classification than current disease entities ^15^. We hope that this influx of advanced molecular diagnostics will ultimately contribute to more personalized tailoring of treatment strategies and translate to improved survival.

## Optimization of current treatment approaches for MF

Great efforts are dedicated to improving the treatment armamentarium currently available for MF. We will focus on what we perceive are the main trajectories within these efforts: development of second-generation JAK inhibitors, advances in transplantation medicine and its utilization for MF, and evaluation of novel agents. Although a complete summary of current treatment approaches is beyond the scope of this review, it is important to note the emerging role for pegylated interferon formulations in the treatment of early stage (proliferative) MF ^23– 25^ and of hypomethylating agents in accelerated and MPN-BP ^26– 28^.

### JAK inhibitors

Following the discovery of *JAK2*V617, a non-selective JAK1/2 inhibitor, ruxolitinib, was developed and has been shown in two pivotal phase III studies to induce significant spleen volume reduction and improvement in constitutional symptoms, leading to its approval by the USA and European regulatory agencies for intermediate-2 and high-risk MF ^29, 30^. The widespread use of ruxolitinib has changed the therapeutic landscape and significantly impacted the quality of life of many MF patients. Moreover, long-term follow up of the COMFORT studies has suggested a survival advantage with the use of ruxolitinib ^31^. Since the biological effects of this drug on mutation allele burden or bone marrow histological findings have been inconsistent and overall modest ^32– 34^, other analyses have attributed the beneficial effect of ruxolitinib on survival to the attenuation of systemic inflammation and reversal of cachexia owing to spleen volume reduction. Furthermore, the use of ruxolitinib is limited in patients with anemia and thrombocytopenia. These considerations inspired the development of second-generation JAK inhibitors that lack myelosuppressive effects and may allow effective treatment for patients with cytopenias. Three second-generation JAK inhibitors have been evaluated in phase III clinical trials, and their efficacy results are summarized in Table 1.

**Table 1.** Summary of the efficacy outcomes of the six larger scale (phase II and III) second-generation JAK inhibitor studies. BAT, best available therapy; DIPSS, Dynamic International Prognostic Scoring System; FED, fedratinib; IPSS, International Prognostic Scoring System; JAK, Janus kinase; MF, myelofibrosis; MOM, momelotinib; PAC, pacritinib; PLT, platelet; pts, patients; RBC, red blood cell; RUX, ruxolitinib; SVR, spleen volume reduction; TSS, total symptom score.

Drug	Trial	Patient population	Number of patients	Comparator	Spleen response (SVR≥35%) at 24 weeks	Symptom response (≥50% reduction in TSS) at 24 weeks	Cytopenia	Ref.
PAC	PERSIST-1 (phase III)	JAK-inhibitor-naïve pts with DIPSS intermediate or high-risk MF, with no exclusion for baseline anemia or thrombocytopenia	327	BAT (2:1) excluding JAK inhibitors	19% in PAC group versus 5% in BAT group *(P <*0.0003)	19% in PAC versus 10% in BAT	25% who were transfusion dependent achieved transfusion independence. Responses for pts with low PLT counts	35
PAC	PERSIST-2 (phase III)	MF pts with PLT counts <100 x 10 ^9^/L	311	BAT (2:1) including RUX (48%)	18% in PAC group versus 3% in BAT group	25% in PAC group versus 14% in BAT group	Reduced transfusion burden in PAC group	36
MOM	SIMPLIFY-1 (phase III)	JAK-inhibitor-naïve adult pts with IPSS intermediate-2 or high-risk MF, or symptomatic intermediate-1 MF	432	Ruxolitinib (1:1) (the only head- to-head study)	26.5% in MOM group versus 29% in RUX group (non-inferior)	28.4% in MOM group versus 42.2% in RUX group (inferior)	66.5% of MOM pts transfusion independent at 24 weeks versus 49.3% with RUX	37
MOM	SIMPLIFY-2 (phase III)	Adult MF patients who had suboptimal responses or hematological side effects with RUX	156	BAT (RUX in 89%) (2:1)	7% in MOM group versus 6% in RUX group (not superior)	26% in MOM group versus 6% in BAT group ( *P* = 0.0006)	RBC transfusion independence at 24 weeks achieved by 43% of MOM group versus 21% of BAT group	38
FED (two doses)	JAKARTA-1 (phase III)	JAK-inhibitor- naïve pts with intermediate-1 to high-risk MF	289	Placebo (2:1)	36% and 40% in FED 400 mg and 500 mg groups, respectively, versus 1% with placebo	36% and 34% for the two doses versus 7% with placebo	Not reported (drug myelosuppressive)	39
FED (400 mg)	JAKARTA-2 (phase II)	RUX resistant or intolerant patients with intermediate or high-risk MF	97	None (single-arm study)	55% (46 of 83 evaluable pts)	26% (23 of 90 evaluable pts)	Not reported (drug myelosuppressive)	40

Pacritinib is an oral multi-kinase inhibitor with specificity for JAK2, FLT3, IRAK1, and CSF1R. The PERSIST-1 study randomized ruxolitinib-naïve patients irrespective of platelet count to either pacritinib at a dose of 400 mg once daily or best available therapy (BAT) excluding ruxolitinib. Spleen and symptom responses were significantly superior in the pacritinib arm. Notably, patients with low platelet counts achieved comparable benefits. Toxicities were overall manageable ^35^. PERSIST-2 recruited only patients with thrombocytopenia and randomized them to pacritinib at one of two doses or BAT including ruxolitinib. Prior use of ruxolitinib was allowed. Pacritinib at both doses was superior to BAT and led to significant spleen and symptom responses, with tolerable myelosuppression ^36^. The development of pacritinib was halted by a clinical hold placed by the FDA due to suspicion of excess cardiovascular deaths in patients treated with the drug. This hold was subsequently removed, and the results of a new study that may hopefully lead to pacritinib’s approval are awaited.

Momelotinib is a JAK1/2 inhibitor with activity against activin receptors believed to contribute to a remarkable anemia response, rendering this drug attractive, especially in the setting of transfusion-dependent anemia. The two phase III SIMPLIFY studies that evaluated momelotinib, despite showing a compelling anemia response, each failed to show superiority or non-inferiority in a primary or key-secondary endpoint ^37, 38^. A more pragmatic study design that would take into account the anemia response and superior tolerability of this drug along with spleen and symptom responses could allow for a better evaluation of this potentially beneficial agent ^41^. Recently, the development of momelotinib was renewed, and a new phase III study is recruiting patients.

Fedratinib is a selective JAK2 inhibitor. The JAKARTA-1 study evaluated two doses of fedratinib versus placebo and showed significantly superior spleen and symptom responses in both treatment arms ^39^. JAKARTA-2 was a single-arm phase II study evaluating fedratinib in patients who were intolerant of or resistant to ruxolitinib ^40^. This was the only clinical trial directly evaluating a second-generation JAK inhibitor as second-line treatment, and responses, especially with regards to spleen volume reduction, were compelling for this patient population. The development of fedratinib was also stalled by a clinical hold placed by the FDA due to a suspected association with Wernicke’s encephalopathy, which was eventually lifted following re-evaluation. The phase III FREEDOM study (NCT03755518) is currently recruiting patients and will hopefully contribute to further development of this drug with apparent potential clinical benefit. Based on the results of the JAKARTA-1 trial, on 16 August 2019, the FDA approved fedratinib for the treatment of adult patients with intermediate-2 or high-risk MF.

### Bone marrow transplantation

The discovery by Fialkow in 1976 that MPNs are a clonal disorder of HSCs ^3^, more recently confirmed at the molecular level by the Weissman laboratory ^4^, led to the hypothesis that MPNs may be cured by HSCT, a procedure which replaces the malignant HSCs of the recipient with healthy ones provided by the donor. However, since the disease also impairs the supportive bone marrow microenvironment, which remains patient derived after transplantation ^42^, this approach was initially received with skepticism. Reports of long-term survival of transplant recipients coupled by histological evidence of resolution of bone marrow fibrosis eventually provided compelling evidence for the curative potential of this approach ^43– 46^. However, HSCT remained limited to a relatively small proportion of patients owing to the older age and frequent comorbidities of most patients and the high non-relapse mortality reported in early studies.

Recently, advances in transplantation medicine and molecular diagnosis are being translated to a consistent improvement in HSCT outcomes in MF ^47– 49^. Several factors contribute to the increased utilization and improved outcomes of HSCT for MF treatment: 1) the use of reduced intensity conditioning (RIC) regimens in older patients, which have demonstrated decreased transplant-related mortality with comparable relapse rates and overall survival ^50– 52^; 2) increased accessibility to HSCT for patients lacking sibling or matched-unrelated donors owing to advances in the use of alternative donors such as haploidentical donors and cord blood ^53– 55^; 3) accumulating data regarding mutational profiles and their prognostic significance allowing earlier and more informed patient selection ^56^; and 4) accumulating experience with the use of ruxolitinib before or after transplant.

HSCT is presently considered the standard of care in eligible patients with intermediate-2 and high-risk disease ^34^. There is lack of consensus regarding its use in earlier stages of the disease predicted to have more prolonged survival. A subset of patients classified as having low/intermediate-1 risk disease may eventually experience rapid progression to advanced MF and shortened survival. The recent advances in linking genetic profiles with risk of progression may improve the prediction of disease course and allow us to identify early stage patients who should be considered for HSCT.

## Evaluation of novel agents

Numerous investigational agents are being evaluated for the treatment of MF alone or in combination with ruxolitinib. These agents were designed with the aim to a) improve anemia, b) deplete the malignant HSCs by targeting molecular alterations downstream of the genetic lesions, c) reduce microenvironmental abnormalities that may synergize with the driver mutations in sustaining proliferation of the malignant HSCs, or d) boost patients’ immune reactions. Comprehensive reviews focusing on investigational agents for the treatment of MF have been published recently ^57, 58^. We will highlight several agents representing the above aims that have shown a strong scientific and preclinical rationale and/or encouraging signs of activity in early phase clinical studies.

### Agents that improve anemia

Activins are members of the transforming growth factor (TGF)-β superfamily that inhibit the differentiation of late-stage erythrocyte precursors in a mechanism independent of erythropoietin and are overexpressed in myelodysplastic syndromes (MDS) and in MF. Two agents that antagonize the activity of activins, sotatercept and luspatercept, have demonstrated efficacy in the treatment of anemia in patients with MDS ^59– 63^. These agents are currently being evaluated in patients with MF and anemia with promising results in early phase studies ^64^.

### Drugs targeting epigenomic alterations found in malignant HSCs

Members of the Bromodomain and Extra-Terminal (BET) family of proteins function as “readers” of histone modification marks, by interacting with acetylated lysine residues on histone tails, and regulate genes that are involved in inflammation and cancer such as *MYC, BCL-2*, and *NF-kB* ^65, 66^. The NF-kB pathway downstream to BET has been shown to be activated in MF via JAK–STAT and other inflammatory cytokine signaling ^67^. In a preclinical study using two MF mouse models, treatment with a BET inhibitor (BETi) resulted in reduction of cytokine production, spleen volume, and bone marrow fibrosis, and these effects were enhanced in combination with ruxolitinib ^68^. These data indicate that drugs that reduce NFκB function and expression of NF-kB target genes might be effective in treating MF ^69^. CPI-610, a BET inhibitor, is currently being evaluated for the treatment of MF patients, alone or in combination with ruxolitinib (NCT02158858).

While most of the above investigational agents as well as JAK inhibitors target signaling pathways involved in myeloproliferation and systemic inflammation, agents directly targeting MF-HSCs are lacking. The development of such agents has been a major challenge in the effort to improve the therapeutic armamentarium for myeloid malignancies. Imetelstat is an inhibitor of telomerase enzymatic activity ^70^. The rationale for its use in MF stems from the findings of enhanced telomerase activity in MPN granulocytes ^71^. A pilot study evaluating the use of single-agent telomerase in 33 patients with intermediate-2 and high-risk MF demonstrated a modest overall response rate (ORR) of 21%; however, in all four patients who achieved a complete response, bone marrow fibrosis was reversed, and a molecular response occurred in three of these patients, suggesting that imetelstat targets the malignant HSCs ^72^. Preclinical studies confirmed these findings by demonstrating direct targeting of MF-CD34 ^+^ cells by imetelstat, reflected by decreased formation of megakaryocytic colonies and by reduced human chimerism in an immune-deficient mouse xenotransplantation model. Both of these effects were selective to cells from MF patients, but not normal individuals, and sustained over time ^73^. Recently, a multicenter phase II study evaluating two doses of imetelstat in a cohort of MF patients that progressed after or were refractory to ruxolitinib treatment has demonstrated an estimated twofold prolongation of survival as compared to retrospective reports of comparable patient populations, warranting further clinical testing ^74^.

### Targeting the inflammatory microenvironment

Several clinical studies have shown that MPN patients express increased levels of inflammatory mediators ^75^, including the key inflammatory cytokine interleukin-8 (IL-8), the plasma levels of which predict adverse clinical outcomes ^76^. The pathogenetic role of these cytokines is also supported by the observation that JAK inhibition can attenuate features of MPNs *in vivo* through the inhibition of cytokine production in mutant and non-mutant cells ^77^. However, there is limited insight on the pathobiological consequence of aberrant cytokine production in MPNs. It is conceivable that specific targeting of some of the cytokines altered in MPNs may lead to better clinical outcomes than those obtained with JAK inhibitors ^77^. An altered cytokine pathway that holds potential clinical interest in MF is the lipocalin-2 (LCN2)/interleukin-8 (IL-8) axis and its down-stream signaling via NF-kB. There is evidence that the sequence of events leading to abnormalities in the microenvironment in MF spleens and marrow includes, at least in part, LCN2 ^78^. LCN2 increased the proliferation of splenic endothelial cells, and LCN2 treatment of splenic stromal cells led to increased elaboration of IL-8, which contributes to the creation of an endothelial cell niche supporting the proliferation of MF-HSCs ^79, 80^. This endothelial cell niche can be disrupted *in vitro* by reparixin, an antagonist of the receptors for IL-8 CXCR1/2 ^81, 82^ that are highly expressed on MF spleen CD34 ^+^ cells ^79^. These data indicate that reparixin may represent a novel therapy which can antagonize the effects of IL-8 and thereby disrupt the HSC niche function of splenic endothelial cells. A clinical trial to test the effects of reparixin in MF is planned to be opened by the Myeloproliferative Neoplasm Research Consortium.

### Boosting the patient’s immune system

An important emerging approach in cancer therapy is activation of endogenous anti-tumor activity by treatment with agents that suppress the immune evasion mechanisms activated by cancer cells ^83^. Checkpoint inhibitors, which block the interaction between programmed cell death (PD)1 and its ligand PD-L1 that is exploited by cancer cells to prevent attack by cytotoxic T-lymphocytes, have shown impressive anti-tumor responses in several solid cancers and B-lymphoid malignancies ^83^. There is a strong pre-clinical rationale for inhibition of PD1/PD-L1 interaction in MF ^84– 86^, and several clinical trials are ongoing (NCT03065400, NCT02421354, and NCT02871323). In addition, recent studies suggest that mutant calreticulin and JAK2 can induce specific anti-tumor T-cell responses, which can be harnessed for the development of mutation-specific peptide vaccines ^87, 88^. These and other immune therapy approaches, such as bispecific antibodies, are still in early stages of development.

## Harnessing the altered p53–TGF-β circuitry to treat MF

Although MF originates at the level of HSCs, the predominance of the malignant HSCs over the reservoir of normal HSCs is sustained by tumor-induced micro-environments of bone marrow and spleen ^89, 90^. Studies in animal models indicate that these microenvironmental abnormalities may induce MF independently from the presence of driver mutations ^91^. It is believed that the interaction between MF and abnormal microenvironments determine, at least in part, the clinical sequelae of MF as well as the rate of progression and evolution to MPN-BP ^75^. In addition to abnormalities described in a previous section, “Targeting the inflammatory microenvironment”, possibly inter-related abnormalities for which promising investigational agents are available are represented by reduced expression of p53 in malignant HSCs and increased TGF-β bioavailability in their supporting microenvironment.

### Targeting the reduced expression of p53 in MF-HSCs

p53 is a tumor suppressor that regulates cell cycle, apoptosis, DNA repair, and senescence of many cell types ^92, 93^. Hypomorphic/loss-of-function mutations of *p53* are associated with tumor progression in most human cancers ^18^, and inactivating mutations are observed in 20% of MPN-BP while deletion of *p53* leads to the development of AML in *JAK2*V617F-harboring mice ^94^. Although in patients with chronic-phase MPN *p53* is usually wild-type, as discussed in “Targeting the inflammatory microenvironment”, malignant HSCs are p53 hypomorphic, as they express low levels of the protein ^95, 96^.

Studies to determine the levels of p53 in MF patients with no obvious *p53* mutations were inspired by Takaoka *et al*., who reported that interferon suppresses tumor progression and activates the immune response by increasing the expression of p53 in mouse models of fibroblast transformation ^97^. Since interferon is an effective therapy in patients with MPNs ^98^, it was hypothesized that interferon is at least partially effective in these patients by increasing the otherwise low levels of p53 expressed by the malignant HSCs ^99^. As in other cancers ^95, 100^, the mechanisms that reduce p53 in MPNs may be represented by overexpression of its major regulators MDM2 (HDM2 in humans) and MDM4 (HDM4/HDMX), which are expressed at levels higher than normal in MF CD34 ^+^ cells ^99^. Overexpression of HDM4 and HDM2 in MF may be genetically determined. The two genes are localized on chromosomes 1 and 12, respectively, and gain of 1q and 12q rearrangements are frequently associated with disease progression in MPNs ^101^. Alternatively, the observation that high TGF-β1 induces the expression of HDM2 in breast cancer driving its late metastatic stage ^102^ suggests that, even in the absence of chromosomal duplication, the great levels of TGF-β expressed in the microenvironment (see following section) may drive high levels of HDM2 in MF.

The existence of a p53/HDM2 cycle in MF is important because binding of p53 to HDM2 resulting in p53 ubiquitination and degradation is inhibited by a class of compounds called Nutlins. Nutlins (Nutlin-3, RG7112, RG7388, HDM201, and KRT232) bind to the MDM2 p53-binding site, interfering with its interaction with p53, leading to p53 accumulation and activation ^95, 100, 103– 106^. Proof-of-principle for the therapeutic use of Nutlins in MF was provided by the observation that the orally available Nutlin RG7388 (idasanutlin) depletes MPN-HSCs in culture ^99^. In addition, low doses of the Nutlin RG7112 induce apoptosis of MF CD34 ^+^ cells and reduce donor cell chimerism and *JAK2*V617F allele burden in NSG mice transplanted with MF CD34 ^+^ cells ^107^. The promising results of an open label phase I study of idasanutlin as a single agent in patients with PV and ET presented by Dr. Mascarenhas *et al*. in 2017 at ASH ^108^ and the observation that in AML the patients with the highest overexpression of HDM2 are most responsive to therapy with idasanutlin ^109^ suggest that MF patients, who express higher levels of HDM2 than those observed in PV, will respond well to idasanutlin. A clinical trial with idasanutlin in MF will be conducted soon by the Myeloproliferative Neoplasm Research Consortium.

### Targeting the TGF-β circuitry in MF

Megakaryocytic hyperplasia was the first histopathological hallmark identified in MF ^110^. These early studies also identified that this abnormality is associated with increased TGF-β content and release due to pathological emperipolesis (the interaction between megakaryocytes and neutrophils) ^111^. However, in spite of the overwhelming evidence linking TGF-β with fibrosis in other systems ^112, 113^, the link between megakaryocyte and TGF-β abnormalities and disease progression in MF has been obscured for some time by the fact that they are not direct targets of the driver mutations.

The link between driver mutations, alterations of megakaryocyte maturation, and disease progression in MF has been recently clarified by the observations that mice expressing *JAK2*V617F only in megakaryocytes develop MF ^114^; the driver mutations induce a ribosomopathy that reduces the content of the transcription factor GATA1 (which is essential for terminal maturation) in megakaryocytes, halting their maturation ^115, 116^; mice lacking the regulatory sequences which specifically drive GATA1 expression in megakaryocytes ( *Gata1* ^low^ mice) develop the same megakaryocyte abnormalities observed in MF and MF with age ^117, 118^; and, finally, treatment with an inhibitor of Aurora kinase A, a protein overexpressed in MF megakaryocytes, rescues GATA1 expression in these cells, curing MF in animal models ^119^, suggesting GATA1 as a druggable target in MF ^120^. This clinical hypothesis was tested by demonstrating that an inhibitor of Aurora Kinase A has some efficacy in MF patients ^121^. Owing to these exciting results, additional megakaryocyte abnormalities are currently being considered as potential therapeutic targets for MF.

The Vainchenker laboratory hypothesized for the first time that TGF-β represents a target to cure MF by demonstrating that malignant HSCs lacking the TGF-β gene fail to induce MF upon transplantation in healthy recipients ^122^. Furthermore, malignant HSCs did not induce MF when transplanted into recipients lacking the TGF-β receptor 1 gene ^123^. These results suggest that TGF-β produced by the progeny of the malignant HSCs exerts its pathobiological effects by activating its receptor on a cell population present in the microenvironment. However, attempts to explore the clinical potential of TGF-β in MF were hampered for some time by the fact that the gene ablation strategy used by Vainchenker is not practical in clinical settings.

**Figure 1.**
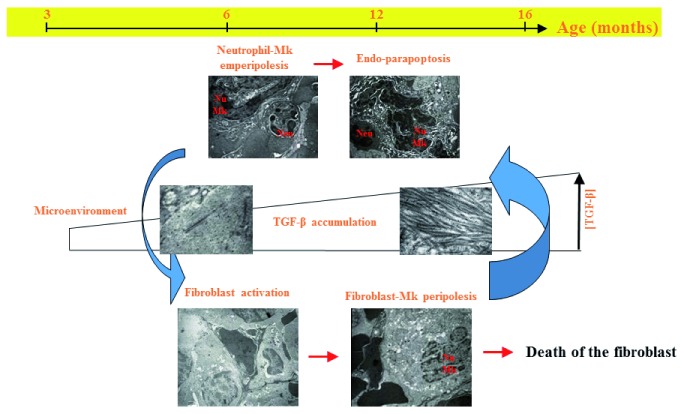
A cellular model for the establishment of increased transforming growth factor (TGF)-β bioavailability, which leads to fibrosis and disease progression in myelofibrosis (MF). This model was elaborated thanks to the fact that, in contrast with other animal models that develop a MPN phenotype that rapidly progress into its fatal MF phase (discussed in 114), *Gata1* ^low^ mice slowly develop MF with age ^117, 118^. From 1–8 months, *Gata1 ^low^* mice express pre-MF traits such as splenomegaly, increased rates of thrombosis, and osteosclerosis. From 8–12 months, they display MF traits including fibrosis and neovascularization, and from 12 months until their natural death they express a late-MF phenotype which includes increased stem/progenitor cell trafficking and extramedullary hematopoiesis in liver. The various phases are characterized by a sequence of abnormal cellular interactions that finally result in increased TGF-β bioavailability in the microenvironment. First, a pathological neutrophil-megakaryocyte (Mk) emperipolesis leads to death of the megakaryocytes by para-apoptosis, which releases TGF-β into the microenvironment. Second, TGF-β activates fibrocytes to produce collagen and to establish contacts with megakaryocytes, leading to the death of additional megakaryocytes and the release of activated lysyl-oxidase 2 (LOX2) into the microenvironment ^135^. LOX2 polymerizes the collagen produced by the activated fibrocytes into collagen fibers, resulting in fibrosis. The collagen fibers are heavy binders of TGF-β, inducing the formation of areas of increased TGF-β bioavailability in the microenvironment (see also 128).

TGF-β is known to play a major role in the development of fibrosis in multiple organs ^124^ and in the induction of tumor-supporting microenvironments in other cancers ^125, 126^. The experiments by Vainchenker do not clarify whether TGF-β promotes MF by inducing fibrosis and therefore by hampering the ability of the bone marrow microenvironment to support normal HSCs and/or by inducing a microenvironment supporting the malignant HSCs in extramedullary sites. Using the *Gata1 ^low^* mouse model, our laboratory has identified at least some of the events linking megakaryocyte abnormalities, increased TGF-β, and development of MF. We determined that, although plasma and washes from bone marrow and spleen of *Gata1 ^low^* mice, as well as those from MF patients, contain levels of total and bioactive TGF-β modestly (twofold) greater compared to normal controls ^127, 128^, the megakaryocytes and their surrounding microenvironment contain levels of TGF-β 1000–2000-fold greater than normal ^128, 129^, suggesting that it is not increased TGF-β content per se but its increased bioavailability that plays a major role in inducing disease progression in MF. By careful electron-microscopy analyses, we also determined that the increased TGF-β bioavailability is established by a sequence of abnormal cellular interactions involving megakaryocytes, neutrophils, and activated fibrocytes that are mediated by adhesion receptors that are druggable ( Figure 1). Ablation of one of them, P-selectin, rescues MF in *Gata1 ^low^* mice, providing clinical rationale for the use of P-selectin inhibitors, alone or in combination with ruxolitinib, in MF.

**Figure 2.**
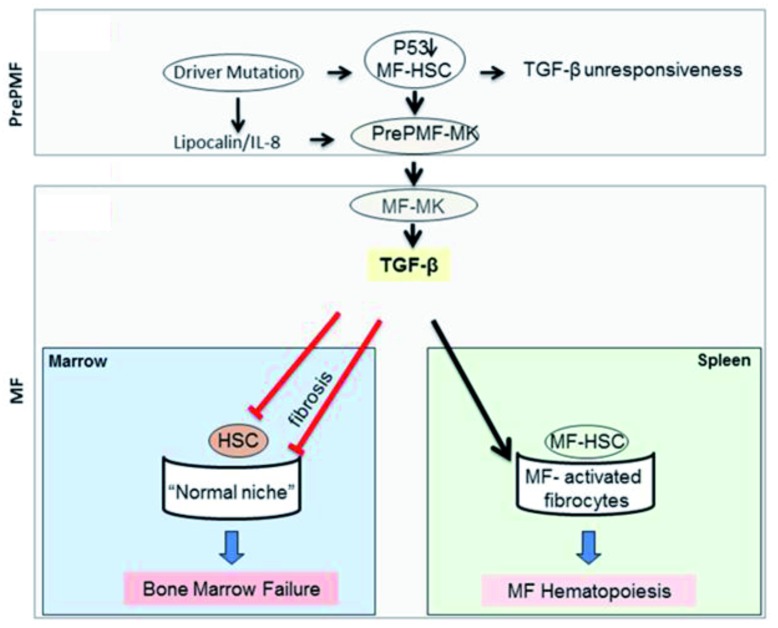
Circuitry between p53 abnormalities in the malignant hematopoietic stem cells (HSCs) and transforming growth factor (TGF)-β in the supporting microenvironment leading to disease progression in myelofibrosis (MF). It is suggested that in MF disease progression is driven by a p53/TGF-β circuitry. In the pre-MF stage, the driver mutations, possibly by inducing an inflammatory milieu (lipocalin-2 [LNC2]/interleukin-8 [IL-8]), reduce p53 in MF-HSCs, making these cells unresponsive to TGF-β and retarding megakaryocyte maturation. Retarded megakaryocyte maturation, associated with high IL-8 expression, induces, in an autocrine fashion, megakaryocytes to increase TGF-β bioavailability, which in turn is responsible for suppressing hematopoiesis from normal HSCs (inducing bone marrow failure) and for promoting an MF-HSC-supporting microenvironment in the spleen, facilitating the transition of pre-MF to MF (modified from 137). MK, megakaryocyte; PMF, primary myelofibrosis

To identify additional druggable TGF-β targets, we characterized the TGF-β expression profiling of bone marrow and spleen from MF patients and *Gata1 ^low^* mice ^128, 130^. These experiments identified that the tissues from the patients and the mouse model express the same distinctive abnormalities which include reduced expression of the canonical SMAD 1, 2, and 4 signaling and increased expression of *JUNB*, *EVI1*, and *STAT1*, three genes downstream of the non-canonical MAPK signaling. These abnormalities predict the activation of non-canonical p38/ERK-dependent rather than canonical SMAD-dependent signaling. The knowledge that activation of SMAD signaling induces normal HSCs into quiescence ^131, 132^ while in systemic sclerosis activation of the p38/ERK pathway promotes the development of fibrosis ^133, 134^ suggests a model for progression from the indolent phase of the disease (pre-MF) to MF depicted in Figure 2. In this model, increased levels of TGF-β promote disease progression by reducing the number of normal HSCs (inducing them into quiescence and reducing their supportive microenvironment in the bone marrow) while increasing the number of malignant HSCs (which are insensitive to TGF-β and are sustained by a specific microenvironment induced by TGF-β in the spleen).

Circumstantial evidence in support of the model for disease progression depicted in Figure 2 exists. First, we observed that the spleen, but not the bone marrow, from MF patients and *Gata1 ^low^* mice contains greater numbers of activated fibrocytes that establish physical contacts with megakaryocytes, forming “nests”, which lodge hematopoietic cells with the morphology of HSCs ^128, 136^. In the mouse model, the presence of these nests is induced by TGF-β. Second, we demonstrated that in contrast to normal HSCs, MF-HSCs are not induced into cycle by treatment with TGF-β receptor 1 kinase inhibitors ^137^.

Based on this overwhelming evidence, a proof-of-principle experiment was conducted that provided findings that one-month treatment with an inhibitor of LK5, the first element of the TGF-β receptor I signaling, completely rescues MF in *Gata1* ^low^ mice ^129, 136^. Furthermore, a limited clinical trial with a human neutralizing antibody against TGF-β (fresolimumab, Sanofi Aventis) provided evidence for sustained improvements in hemoglobin level ^137, 138^ and of reduced bone marrow fibrosis that lasted for at least one year after the drug was discontinued ^137^ in at least two of the three patients treated. A drawback of drugs targeting TGF-β is their lack of specificity that makes them prone to off-target effects. The availability of ligand-traps specific for TGF-β1, such as AVID200 developed by Formation Biologics ^139^, makes conducting clinical trials to test the efficacy of TGF-β inhibition in MF and eventually to halt the progression from pre-MF to MF in patients without fear of cardiovascular or osteological complications finally possible. AVID200 will be tested in a clinical trial to be opened soon by the Myeloproliferative Neoplasm consortium.

The existence of a circuitry between p53 and TGF-β in development and cancer progression has been extensively discussed ^140– 142^. In addition to the possibility that high levels of TGF-β may be responsible for reducing p53 in cancer cells by inducing HMD2 discussed in a previous section, “Targeting the reduced expression of p53 in MF-HSCs” ^102^, other feedbacks between the two pathways have also been described. In some cell models, low levels of p53 are responsible for rendering malignant cells insensitive to the inhibitory effects of TGF-β on proliferation ^143^. It is therefore possible that, also in MF, low levels of p53 make the malignant HSCs TGF-β unresponsive. This consideration suggests that combination therapies targeting p53 (Nutlins) and TGF-β (AVID200), by disrupting the cross-talks between the two pathways, have a greater potential than single therapies to cure MF. This hypothesis will be tested by ongoing preclinical studies.

## Lessons learned from studying MF are broadly applicable to cancer pathogenesis and therapy

As previously discussed, the dissemination of malignant hematopoiesis beyond the marrow occurring during MF progression is associated with the establishment of malignant HSC-supporting microenvironments in the spleen. These events are strikingly similar to those occurring in many metastatic solid tumors ^125, 126^, suggesting that lessons learned from studying MF may be applied to studying the progression of numerous other tumor types.

Induction of tumor-specific microenvironments as well as cell trafficking are complex processes that involve hundreds of genes, many of which are implicated in multiple biological processes. A comparison of gene dysregulation in *Gata1 ^low^* mice and in the metastatic phase of other solid cancers provides support for the hypothesis that the two pathologies are determined by common mechanisms. Pathway analyses of expression profiling of bone marrow and spleen from *Gata1 ^low^* mice identified two altered expression signatures involving 20–35 genes of the TGF-β1 signaling pathway with important hematopoietic functions ^129^. In bone marrow, there was significant down-regulation of *Bmp2*, *Bmp5*, *Acvrl1*, and *Igf1*, whereas in the spleen, there was significant overexpression of *Cdkn1a* and *Ltbp1* and underexpression of *Gdf2* and *Nodal*. Interestingly, several of these events are reported also in other metastatic solid tumors. For example, TGF-β is dysregulated in colorectal cancer with similarity to those noted in the bone marrow, including downregulation of *Bmp2* and *Bmp5* ^144^; ACVRL1 overexpression is also observed in colorectal cancer, where it has been suggested as a prognostic biomarker for the metastatic phase ^145^; and TGF-β and its signaling crosstalk play a crucial role during the endothelial mesenchymal transition, which promotes the metastatic phase of various solid tumors, particularly in breast and pancreatic carcinoma ^146^. These similarities strongly suggest that lessons learned from studying MF can be applied to studying the pathogenesis of other tumor types.

Finally, fibrosis is the end stage of all organ failures, including liver, kidney, heart, and lung, which, when associated with MF, have poor prognosis. It is conceivable that the identification of treatments which prevent or delay the progression of MF to PMF may inspire treatments for other organ failures as well.

## Conclusion

Although MPNs are uniformly associated with the activation of the JAK/STAT signaling pathways, therapy with currently available JAK2 inhibitors is unable to deplete or eliminate MPN-HSCs. New developments in our knowledge on the biology of MPNs have identified an interplay among HSCs and microenvironmental abnormalities that may sustain disease progression. This cycle is druggable by BETi, CXCR1/2 inhibitors, p53 activators, and TGF-β inhibitors. These drugs are currently the subject of careful investigation in preclinical models and clinical studies in MF patients as single agents. We foresee that these careful approaches will soon identify the best agents that, alone or in combination, may take us closer to the cure of MF in the near future.
